# Biodistribution of Amikacin Solid Lipid Nanoparticles after Pulmonary Delivery

**DOI:** 10.1155/2013/136859

**Published:** 2013-08-01

**Authors:** J. Varshosaz, S. Ghaffari, S. F. Mirshojaei, A. Jafarian, F. Atyabi, F. Kobarfard, S. Azarmi

**Affiliations:** ^1^Department of Pharmaceutics, Faculty of Pharmacy and Drug Delivery Systems Research Center, Isfahan University of Medical Sciences, P.O. Box 14155-6451, Isfahan, Iran; ^2^R&D and Quality Control Department, Iranian Parenteral and Pharmaceutical Co., P.O. Box 18735-568, Tehran, Iran; ^3^Department of Medicinal Chemistry, Faculty of Pharmacy, Tehran University of Medical Sciences, P.O. Box 14155-6451, Tehran, Iran; ^4^Department of Pharmacology and Toxicology, Faculty of Pharmacy, Isfahan University of Medical Sciences, P.O. Box 14155-6451, Isfahan, Iran; ^5^Department of Pharmaceutics, Faculty of Pharmacy, Tehran University of Medical Sciences, P.O. Box 14155-6451, Tehran, Iran; ^6^Department of Medicinal Chemistry, Faculty of Pharmacy and Pharmaceutical Sciences, Shahid Beheshti University of Medical Sciences, P.O. Box 14155-6153, Tehran, Iran; ^7^Faculty of Pharmacy and Pharmaceutical Sciences, University of Alberta, Edmonton, AB, Canada T6G 2E1

## Abstract

The main purpose of the present work was studying the biodistribution of amikacin solid lipid nanoparticles (SLNs) after pulmonary delivery to increase its concentration in the lungs for treatment of cystic fibrosis lung infections and also providing a new method for clinical application of amikacin. To achieve this aim, ^*99m*^Tc labelled amikacin was loaded in cholesterol SLNs and after *in vitro* optimization, the desired SLNs and free drug were administered through pulmonary and *i.v.* routes to male rats and qualitative and biodistribution studies were done. Results showed that pulmonary delivery of SLNs of amikacin by microsprayer caused higher drug concentration in lungs than kidneys while *i.v.* administration of free drug caused reverse conditions. It seems that pulmonary delivery of SLNs may improve patients' compliance due to reduction of drug side effects in kidneys and elongation of drug dosing intervals due to the sustained drug release from SLNs.

## 1. Introduction

Cystic fibrosis (CF) is the most common lethal hereditary disorder with autosomal recessive heredity in the Caucasian population. This disease is characterized by extremely high viscosity secretions from exocrine glands in the airways. The increased viscosity of mucus leads to a reduced clearance of microorganisms from the respiratory tract and chronic bacterial infection of the airways. 

Amikacin is an aminoglycoside which is active against most gram-negative bacteria and is used for the treatment of cystic fibrosis infections. This antibiotic is used in high concentrations to reach the therapeutic levels in the lungs which results in serious nephrotoxicity side effects.

 A drug delivery system that helps to increase the therapeutic index of the aminoglycosides by increasing the concentration of drug at the site of infection and/or reducing the nephro- and ototoxicity would attract considerable interest. 

Many delivery systems have been investigated so far to reduce these toxicities such as liposomal amikacin dry powder inhaler [1], intratracheal delivery of gentamycin [2], high dose nebulization of amikacin [3], and DPI formulation for cystic fibrosis patient [4]. Solid lipid nanoparticles (SLNs) of amikacin were designed, optimized, and characterized *in vitro* by Varshosaz et al. [5], but there is no published data on* in vivo *study on pulmonary delivery of SLNs of amikacin.

Pulmonary delivery is considered as an alternative route for local drug delivery. The potential advantages of direct delivery of drug to the lung infection diseases include the possibility of reduced systemic toxicity, as well as achieving higher drug concentration at the main site of infection. Particles in the size range of 100–500 nm can be successfully deposited into various regions within the respiratory tract when they are incorporated into suitable vehicles, such as aerosols or dry powders, and it is anticipated that in the near future pulmonary diseases may be treated using inhalable therapeutic agents loaded into nanoparticles [6]. There were numerous studies published about the pulmonary delivery of colloidal carriers in recent years. Examples of drugs for pulmonary delivery using colloidal carriers are amiloride hydrochloride and secretory leukocyte protease inhibitor (for cystic fibrosis), tobramycin, rifampin, ciprofloxacin, itraconazole, leuprolide, and doxorubicin for lung cancer [7].

The objective of the present study was to investigate the biodistribution of SLNs of amikacin after pulmonary delivery and possibly the increase of drug concentration in the lungs compared to other organs like kidneys which are the main organ affected by the side effects of amikacin. For this purpose *in vivo* behavior of SLNs of amikacin in rats after *i.v.* and pulmonary administration was investigated. 

## 2. Materials and Methods

### 2.1. Materials

Cholesterol, Tween 80, ethanol, acetone, potassium hydrogen phosphate, sodium hydroxide, and SnCl_2_ were purchased from Merck (Germany). Ketamine was from Alfasan (The Netherlands). ^*99m*^Tc pertechnetate was supplied as ^*99*^MO/^*99m*^Tc generator and amikacin was purchased from Hangzhou Uniwise International Co. (China).

### 2.2. ^*99m*^Tc-Amikacin Radio-Labeling Procedure

50 mg of amikacin was dissolved in 3 mL of PBS (0.5 M, pH 7.4), and then 1.1 mg of SnCl_2_ in 275 *µ*L HCl 0.5 M was added into the vial. The vial was reconstituted with 1850 MBq of sodium pertechnetate mixed with 1 mL of saline and the mixture was incubated at room temperature for 15 minutes. 

### 2.3. Radiochemical Analysis of  ^*99m*^Tc-Amikacin by RTLC

The labeling yield and radiochemical purity were determined by thin layer chromatography. The reaction product was spotted on silica gel ITLC-SG strips (Sigma Chemical Company, USA), 10 × 1.5 cm^2^ sheets, and developed in acetone and triple solvent including ethanol/ammonia/water 2 : 1 : 5 as the mobile phase. After developing, they were cut into 1 cm pieces and counted with a NAI (T1) detector equipped with a single channel analyzer. 

In this system, free pertechnetate was moved away with solvent front leaving ^*99m*^Tc and reduced hydrolyzed technetium at the point of application. Reduced hydrolyzed technetium was separated using solvent mixture (ethanol/ammonia/water: 2 : 1 : 5) as mobile phase in which free pertechnetate and ^*99m*^Tc-amikacin moved with solvent front whereas reduced hydrolyzed technetium remained at the point of application.

The radiochemical purity (RP) or labeling efficiency was determined using (1), in which *cpm* is *count per minute *[8]:

(1) RP%=cpm  of  Tc99m-Amikacin∑cpm  (Tc99mO2+Tc99mO4+Tc99m-Amikacin).

### 2.4. Stability of  ^*99m*^Tc-Amikacin

After labeling of amikacin with ^*99m*^Tc (^*99m*^Tc-amikacin), it was left at room temperature for two, four, six, and twelve hours. The labeling stability of ^*99m*^Tc-amikacin was evaluated by determination of the radiochemical purity after each time point (*n* = 3). 

### 2.5. Preparation of SLNs of Amikacin

The SLNs of amikacin were prepared as reported previously [5] to achieve particles with optimum particle size and drug loading efficiency. Briefly, 50 mg of amikacin or ^*99m*^Tc-amikacin in PBS (0.5 M, pH 7.4) was dissolved in deionized water containing 1% w/w Tween 80 to reach maximum 50 mL in volume and homogenized at 11000 rpm (IKA T-18 basic, Ultraturrax, Germany). 150 mg of cholesterol as lipid phase was dissolved in ethanol/acetone mixture with the ratio of 3 : 1 by heating to 70°C and stirring. Then hot oily phase was added to aqueous phase in 25°C under homogenization at 11000 rpm. The prepared emulsion was sonicated in bath sonicator (Ultrasonic system, Tecna 6, Tecno Gaz, Italy) and cooled to room temperature to achieve nanoparticles.

### 2.6. Particle Size and Zeta Potential

Particle size and zeta potential of SLNs were measured using Malvern Zetasizer (Nano ZS3000; Malvern, UK).

### 2.7. Drug Loading Efficiency

Loading efficiency of amikacin in SLNs was calculated by (2) [9] using indirect method. In this method nanoparticle dispersion was centrifuged (Sigma, Germany) at 35000 rpm for 45 min at −4°C. The concentration of amikacin in supernatant was analyzed by derivatization HPLC method [5]:

(2) Drug  loading  efficiency  % =Drugtotal−DrugsupernatantDrugtotal×100.

### 2.8. Drug Release Study

Drug release study was performed using dialysis sack method by DO405 Dialysis tubing 23 × 15 mm with 10–12 kD cut-off (Sigma, USA). Five mL of the optimized formulation was placed in dialysis membrane and the sack was immersed in 50 mL of phosphate buffer solution (pH 7.4). One mL sample was withdrawn at predetermined time intervals and drug concentration was analyzed using precolumn derivatization by HPLC method [5].

### 2.9. Morphology Study

Morphology of the nanoparticles was characterized by scanning electron microscopy (SEM). The nanoparticles were mounted on aluminum stabs, sputter-coated with a thin layer of Au/Pd, and examined using an SEM (Philips XL30, Almelo, The Netherlands).

### 2.10. Animal Studies

Male Wistar rats, body weight 180–200 g, from animal house of the School of Pharmacy and Pharmaceutical Sciences of Isfahan University of Medical Sciences were used for the *in vivo* studies. The animals were housed in colony cages with free access to standard chow pellets and water, under uniform housing in the environmentally controlled conditions (22 ± 2°C, 12 h light-dark cycle, 55–65% humidity), and placed in the laboratory for 4–6 days during the acclimatization period and during the course of the study. The animal study was approved by the guideline of the ethical committee of Isfahan University of Medical Sciences.

### 2.11. Drug Dosing Methods

Eight groups of male Wistar rats (180–200 g) were studied. The first group received SLNs of radio-labeled amikacin via pulmonary route (group A), the second group received free radio-labeled amikacin pulmonary (group B), the third group received *i.v.* SLNs of radio-labeled amikacin (group C), and the fourth group was administered *i.v.* free radio-labeled drug (group D); in each of these groups 9 rats were studied and in each time point (0.5, 2, and 6 hours) three of them were euthanized to remove organs for measuring drug concentration. In these four groups, blood samples were taken from three rats that were killed after 6 hours; blood samples were taken from tail vein in desired time points (5, 15, 60, 120, 180, 240, and 360 minutes after *i.v.* or pulmonary drug administration). A catheter was inserted in the tail vein for *i.v.* drug administration and blood sample collection. Groups E and F were control groups that received blank SLNs by pulmonary and *i.v.* routes, respectively. Groups G and H were two other control groups which received nonradio-labeled free amikacin solution through *i.v.* and pulmonary routes to rule out the possible interaction in drug detection method. Three animals were included in each control group and one of them was killed in each desired time point to remove organs for examination of drug concentration.

The rats in each subgroup were killed using CO_2_ gas in the desired time point to detect drug concentration; before that gamma scintigraphy was done to provide direct image of radio-labeled drug in whole animal bodies.

Rats received 100 *µ*L of SLNs of amikacin or free amikacin solution in equal doses by *i.v.* or pulmonary delivery. For *i.v.* groups tail vein was used to inject drug SLNs or solution and for pulmonary route, 1A_1B microsprayer (Penncentury, USA) was used. Each animal received 100 *µ*L of *i.v.* ketamine solution (10%) before drug administration. 

### 2.12. Gamma Scintigraphy Analysis

*In vivo* evaluation of pharmaceutical products was achieved by *γ*-scintigraphic imaging of the particles deposited in the lung. Imaging provided direct information on the amount and location of the drug deposited in lung after drug administration. For all studies a single headed camera (PICKER-PRISM 1000, USA) employing a low energy gamma high resolution collimator was used.

### 2.13. Quantitative Analysis

Quantitative gamma counting was performed on ORTEC model 4001 M *γ*-system well counter. Lungs, kidneys, stomach, spleen, intestine, and liver of animals were removed for biodistribution studies. Also blood samples were studied to evaluate which groups show the least trough level blood concentration. The drug concentration in lungs and kidneys was compared in each group and between different groups to investigate if SLNs could decrease amikacin concentration in kidneys and if pulmonary delivery could increase drug concentration in lungs to improve treatment of lung infection in severe conditions like infections by *Pseudomonas aeruginosa* in cystic fibrosis.

Equation (3) [10, 11] was used to calculate drug concentration in each organ, in which *ID* meant injected dose and *pure M* was pure weight of each organ(s) as follows:

(3) $$\begin{array}{c} \%\frac{\text{ID}}{\text{gr Organ}}=\frac{\text{eac}{\text{h}}_{\text{count}}}{\text{tota}{\text{l}}_{\text{count}}}\times \frac{100}{\text{pure}\;M}. \end{array}$$

Comparison of drug concentration in different groups also lung and kidney of each group was done by two-way ANOVA followed by Duncan's post hoc test using SigmaPlot software (Version 11, USA).

## 3. Results

Radiochemical analysis of ^*99m*^Tc-amikacin was done by Radio Thin Layer Chromatography (RTLC).

Quality control was done by paper chromatography. In brief, silica gel paper was used as stationary phase and acetone was used as mobile phase for separation of free pertechnetate. A mixed solution including ethanol/ammonia/water in ratio of 2 : 1 : 5 was used as mobile phase to separate reduced hydrolyzed technetium. The maximum radio-labeling yield of amikacin was >98.2% and it was stable at least for 12 hours.

### 3.1. Stability of  ^*99m*^Tc-Amikacin

Radio-labeled amikacin was stable for 12 hours with more than 97 ± 1% radio-labeling yield.

### 3.2. Particle Size

**Figure 1:**
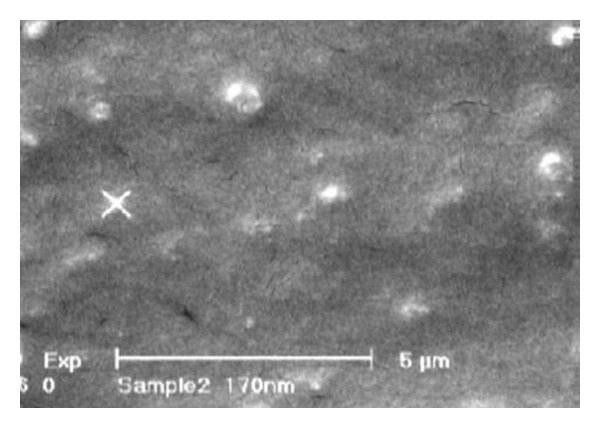
SEM photograph of SLNs of amikacin.

Particle size of desired SLNs was 164 ± 7 nm and the shape of particles was spherical.  Figure 1 shows SEM micrographs of particles.

### 3.3. Drug Loading Efficiency

**Figure 2:**
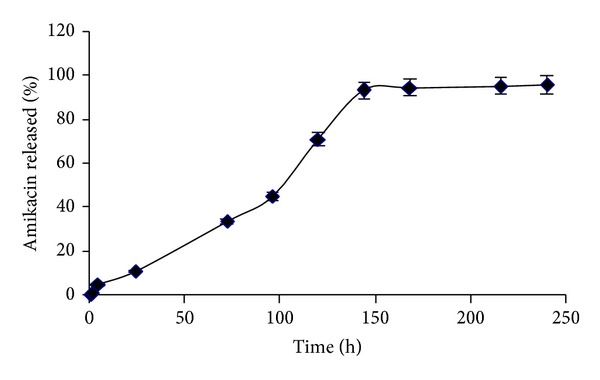
*In vitro* amikacin release profile from SLNs.

Drug loading efficiency was 89 ± 6% and *in vitro* drug release profile was sustained and continued for 144 hours (Figure 2).

### 3.4. Gamma Scintigraphy Analysis

**Figure 3:**
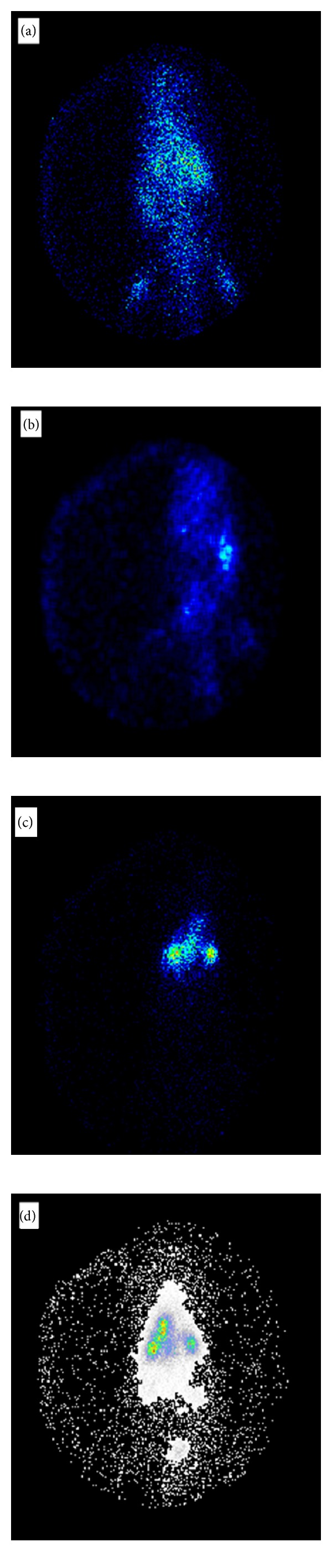
Gamma scintigraphy photographs of rats receiving amikacin loaded SLNs (a) *i.v.* after 0.5 hour, (b) *i.v.* after 6 hours, (c) pulmonary after 0.5 hour, and (d) pulmonary after 6 hours.

Figures 3(a), 3(b), 3(c), and 3(d) show gamma scintigraphy pictures of animals which received ^*99m*^Tc-amikacin SLNs by *i.v.* and pulmonary method after 0.5 and 6 hours of administration, respectively. These figures confirm that SLNs of amikacin remained longer in lungs after pulmonary administration compared to *i.v.* route. Also drug concentration in lungs lasted longer versus other organs when comparing pulmonary and *i.v.* routes. After *i.v.* administration the SLNs were distributed in almost all organs, but after pulmonary administration the drug was concentrated more in lungs, even after 6 hours of administration.

### 3.5. Quantitative Analysis

**Figure 4:**
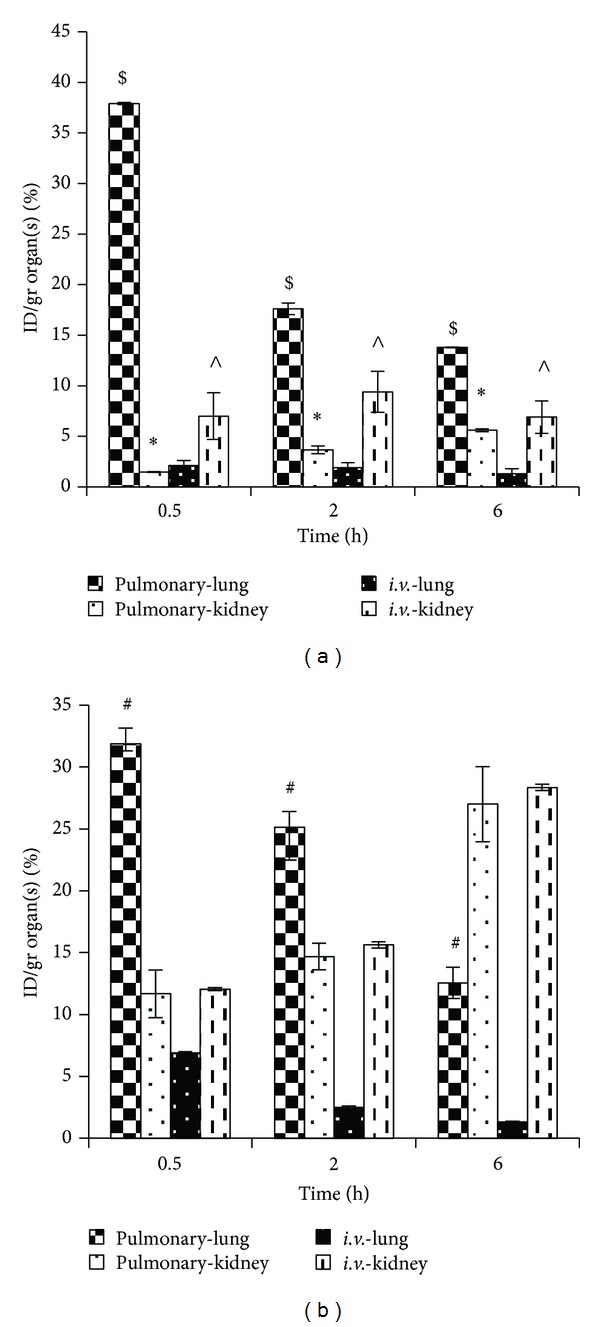
Amikacin concentration in lungs and kidneys after its *i.v.* and pulmonary administration as (a) SLNs and (b) free drug (*n* = 3, *P < 0.05, differences between different times in lung concentrations are significant after pulmonary administration of SLNs; ^$^ *P* < 0.05, lung concentrations in each time are significantly different from kidney concentration after pulmonary administration of SLNs; ^∧^ *P* < 0.05, kidney concentrations are different from lung concentration after *i.v.* administration of SLNs; ^#^ *P* < 0.05, differences in lung concentration are significant between *i.v.* and pulmonary routes).

After *i.v.* administration of drug the two-way ANOVA test was used for comparison of drug concentration in lungs at different time points. Data analysis in group A receiving *i.v.* SLNs of ^*99m*^Tc-amikacin showed no significant difference between drug concentration in lung in different time points (*P* > 0.001) (Figure 4(b)), but in the second group taking *i.v.* free ^*99m*^Tc-amikacin, differences in lung concentration at different time points were significant and reduced by time (*P* < 0.05) (Figure 4(a)).

Analysis of data showed no significant differences between studied time points in the kidneys in the group taking pulmonary free ^*99m*^Tc-amikacin (*P* > 0.05) (Figure 4(a)). It means that drug concentration in kidneys after pulmonary delivery of free ^*99m*^Tc-amikacin was constant and did not change during time. However, pulmonary delivery of ^*99m*^Tc-amikacin SLNs showed gradual increase in drug concentration in kidneys at different time points (*P* < 0.001) (Figure 4(a)).

Figure 4(a) also shows that after pulmonary delivery of amikacin SLNs drug concentration in lungs was significantly higher than *i.v.* method in all the 3 measured time points. Also after pulmonary delivery of amikacin SLNs drug concentration in kidneys was very lower than lungs (*P* < 0.05). Drug concentration in kidneys after pulmonary delivery of SLNs of amikacin was lower than their *i.v.* administration until 2 hours (*P* < 0.05). However, after 6 hours the differences between kidney concentration in *i.v.* and pulmonary routes were not significant (*P* > 0.05).

Figure 4(b) shows that *i.v.* administration of free amikacin caused the same kidney concentration as its pulmonary delivery. However, in each of these methods the drug concentration in kidneys increased during the time (*P* < 0.05). The drug concentration in lungs after pulmonary administration was higher than *i.v.* method (*P* < 0.05). Also by *i.v.* injection of SLNs of amikacin (Figure 4(a)), drug concentration in kidneys was higher than drug concentration in lungs (*P* < 0.05).

**Figure 5:**
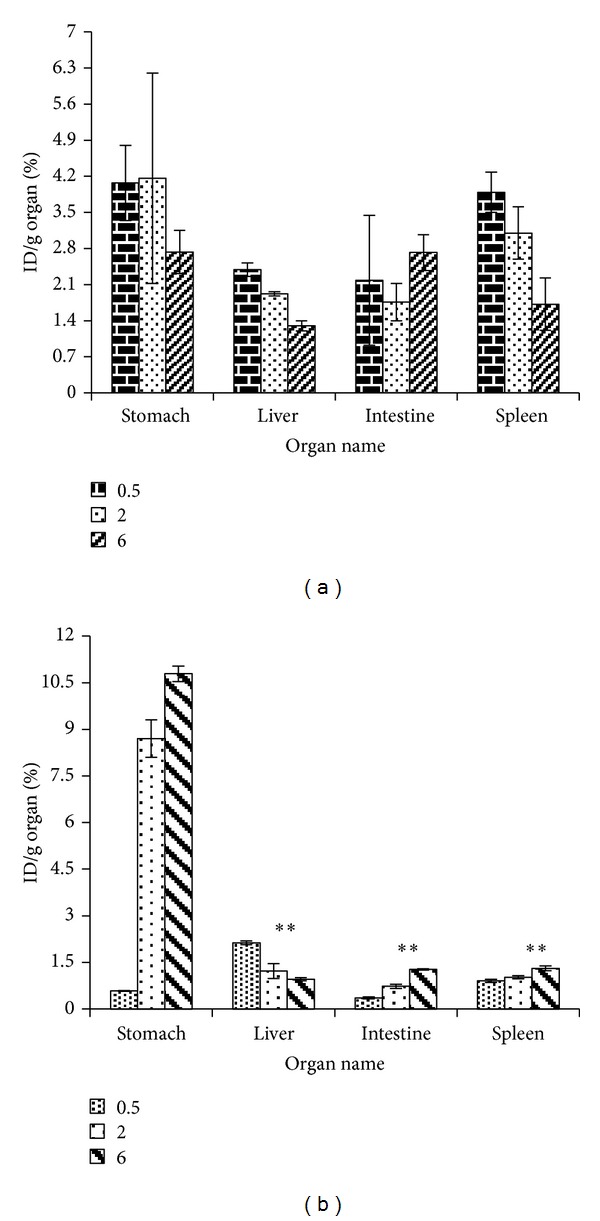
Amikacin concentration in spleen, liver, stomach, and intestine after (a) *i.v.* and (b) pulmonary administration of SLNs (*n* = 3, *drug concentrations in liver, intestine, and spleen are significantly different from stomach at 2 and 6 h after administration *P* < 0.05).

Figures 5(a) and 5(b) show drug concentrations in spleen, liver, stomach, and intestine after *i.v.* and pulmonary administration of SLNs of amikacin in studied time points (0.5, 2, and 6 hours). As it can be seen in these figures, the drug concentration in stomach was higher after pulmonary delivery, which can be related to swallowing of exhaled particles after pulmonary delivery.

**Figure 6:**
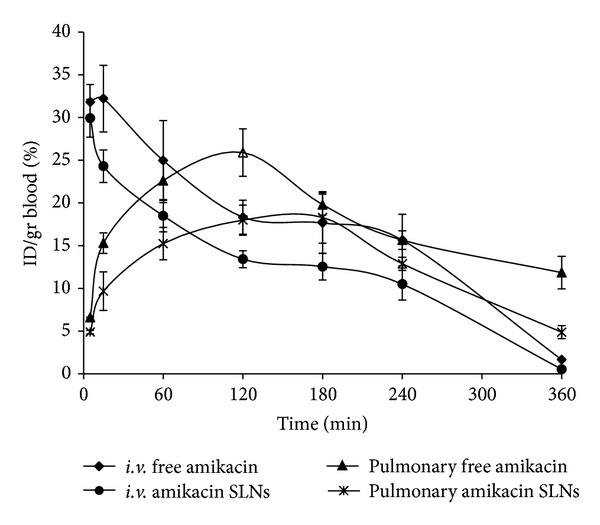
Blood concentration profiles of amikacin after *i.v.* and pulmonary administration of free drug and amikacin SLNs (*n* = 3).

Figure 6 shows drug concentration in blood after *i.v.* and pulmonary administration of free and SLNs of amikacin. It shows that, after pulmonary administration of SLNs of amikacin, the trough concentration of drug which is responsible for auto- and nephrotoxicity of amikacin was lower than pulmonary administration of free drug (*P* < 0.05). Also after *i.v.* administration, drug concentration in blood was higher for free drug than for SLNs almost in all time points (*P* < 0.05). 

## 4. Discussion

This study was designed to evaluate biodistribution of SLNs of amikacin after pulmonary delivery. Some other studies were done to reduce the side effects of drugs by their targeting to the site of infection in diseases like cystic fibrosis [6]. In the present study amikacin was selected as an antibiotic of choice in the treatment of lung infection in cystic fibrosis. In this disease high aminoglycoside doses are needed which results in side effects specially nephrotoxicity. This shows the necessity of using a suitable drug delivery system to decrease the drug toxicity by locally delivering the drug to the site of action. To reach this goal, SLNs of amikacin were prepared with proper size to control drug release and capable to reach the deep alveolar areas of the lungs. As Zetasizer and SEM pictures confirmed the optimized SLNs had suitable size (<200 nm) and zeta potential (+7 mV) to escape from being uptaken by the alveolar macrophages [5, 6, 12]. As reported by Verma et al. [13] inhalation of microparticles (the lager particles than nanometric sizes) or intravenous administration of free isoniazide and rifabutin to mice showed the biodistribution of drugs to the lungs in general and alveolar macrophages in particular.

The optimized amikacin SLNs and free drug were administered to animals by pulmonary and *i.v.* methods to compare the drug biodistribution in these routes and also to investigate the potential of SLNs in reducing the kidney concentration of amikacin and possibly increasing drug concentration in the lungs. Weers et al. [14] followed deposition and clearance of inhaled amikacin-loaded liposomes in healthy male volunteers using gamma scintigraphy. They labeled lipid used in the structure of liposomes while, in our study, for *in vivo* studies amikacin was prelabeled by ^*99*^Tc that let us follow the free and loaded drug in each time.

As expected the concentration of amikacin in lungs after pulmonary delivery of SLNs was more than *i.v.* route. ID/gr organ at 0.5 hour after administration via lung and *i.v.* was 37.9 and 2.1, respectively, and at the last studied time point (6 hours after administration) these were 13.8 and 1.3 which shows clearance of drug from lungs in both methods but confirms our hypothesis about advantages of using SLNs by pulmonary method as a noninvasive method versus *i.v.* route. Although the drug concentration in lungs is higher after pulmonary administration than *i.v.* route, Jain et al. [15] studies showed that intravenous administration of rifampicin solution for effective treatment of pulmonary tuberculosis resulted in a high concentration of drug in serum while it was much lower in the case of PLGA nanoparticles. Coupling of the nanoparticles with lactose significantly enhanced the lung uptake of drug, which caused a higher percentage of doses recovered from the lungs compared to that of the uncoupled drug loaded in nanoparticles and plain drug solution [15]. Wang et al. [16] studies also showed administration of azithromycin liposomes and free azithromycin solution injected intravenously to mice caused slower clearance, increased half-life, and the AUC increased by 7.4-fold in lung by liposomes compared to drug solution. The biodistribution studies and lung-targeting efficiency of dexamethasone SLNs and its solutions in mice after intravenous administration also showed 17.8-fold larger area under the curve of dexamethasone SLNs was achieved compared to that of its solution [17]. However, in spite of all these reports on enhancement of lung concentration of different drugs after *i.v.* administration of different nanosized carriers compared to free drugs, none of them have studied their systems via pulmonary route.

As shown in Figure 4(a) an obvious advantage of pulmonary delivery of amikacin nanoparticles is lower drug concentrations in kidneys that may decrease the probability of nephrotoxicity. However, pulmonary delivery of SLNs caused lower drug concentrations in kidneys compared to pulmonary delivery of free drug. *i.v.* administration of drug loaded SLNs also caused less drug concentration in kidneys compared to free drug by *i.v.* method. Unlike SLNs that did not cause such distribution to spleen and liver (Figure 5), the administration of amikacin in loaded erythrocytes in rats has led to significant changes in the pharmacokinetic behavior of the antibiotic, a greater accumulation being observed in reticuloendothelial organs such as liver and spleen [18]. The relative uptake of amikacin when carrier erythrocytes were used was as follows: spleen > peritoneal macrophages > liver > lung > renal cortex > renal medulla [19].

Following *i.v.* administration of free drug, the drug concentration in kidneys was higher than the lungs (Figure 4(b)). However, drug concentration in the lungs was higher than kidneys following pulmonary delivery of the nanoparticles of amikacin (Figure 4(a)), which is an ideal result to decrease the nephrotoxic side effects and increase the effectiveness of the drug in lungs. The pulmonary administration was reported as a suitable method for targeting of drug liposomes by Weers et al. [14] too. Xiang et al.'s studies [20] showed that *i.v.* administration of dexamethasone SLNs targeted the drug to lungs and reduced systemic side effects while concentrating the drug in lungs to treat local inflammations in asthma and pulmonary fibrosis. 

As reported by Videira et al. [21] an important and significant uptake of the radio-labeled SLNs happened to the lymphatics after inhalation and a high rate of distribution in periaortic, axillar, and inguinal lymph nodes. Results of their study indicate that SLNs could be effective colloidal carriers for lymphoscintigraphy or therapy upon pulmonary delivery. Consequently, the results of our study also confirm that pulmonary use of SLNs of amikacin seems promising for enhanced effects of this drug in cystic fibrosis as inhaled nanoparticles can reduce dose frequency and improve the pharmacologic index of the drug. The further step of this study may be production of a suitable pulmonary dosage form of these nanoparticles and study of their effectiveness in reducing the side effects of amikacin. 

## References

[1] Shah SP, Misra A (2004). Liposomal amikacin dry powder inhaler: effect of fines on *in vitro* performance. *AAPS PharmSciTech*.

[2] Cullen AB, Cox CA, Hipp SJ, Wolfson MR, Shaffer TH (1999). Intra-tracheal delivery strategy of gentamicin with partial liquid ventilation. *Respiratory Medicine*.

[3] Ehrmann S, Mercier E, Vecellio L, Ternant D, Paintaud G, Dequin P-F (2008). Pharmacokinetics of high-dose nebulized amikacin in mechanically ventilated healthy subjects. *Intensive Care Medicine*.

[4] Pilcer G, Goole J, van Gansbeke B (2008). Pharmacoscintigraphic and pharmacokinetic evaluation of tobramycin DPI formulations in cystic fibrosis patients. *European Journal of Pharmaceutics and Biopharmaceutics*.

[5] Varshosaz J, Ghaffari S, Khoshayand MR, Atyabi F, Azarmi S, Kobarfard F (2010). Development and optimization of solid lipid nanoparticles of amikacin by central composite design. *Journal of Liposome Research*.

[6] Gill S, Löbenberg R, Ku T, Azarmi S, Roa W, Prenner EJ (2007). Nanoparticles: characteristics, mechanisms of action, and toxicity in pulmonary drug delivery—a review. *Journal of Biomedical Nanotechnology*.

[7] Mansour HM, Rhee Y-S, Wu X (2009). Nanomedicine in pulmonary delivery. *International Journal of Nanomedicine*.

[8] Sampson CB (1999). *Text Book of Radiopharmacy Theory and Practice*.

[9] Gazori T, Khoshayand MR, Azizi E, Yazdizade P, Nomani A, Haririan I (2009). Evaluation of Alginate/Chitosan nanoparticles as antisense delivery vector: formulation, optimization and *in vitro* characterization. *Carbohydrate Polymers*.

[10] Capriles PV, Dias AP, Costa TE (2002). Effect of eggplant (Solanum melongena) extract on the *in vitro* labeling of blood elements with technetium-99m and on the biodistribution of sodium pertechnetate in rats. *Cellular and Molecular Biology*.

[11] Valenca SS, Lima EAC, Dire GF, Bernardo-Filho M, Porto LC (2005). Sodium pertechnetate (Na^99m^TcO4) biodistribution in mice exposed to cigarette smoke. *BMC Nuclear Medicine*.

[12] Sham JO-H, Zhang Y, Finlay WH, Roa WH, Löbenberg R (2004). Formulation and characterization of spray-dried powders containing nanoparticles for aerosol delivery to the lung. *International Journal of Pharmaceutics*.

[13] Verma RK, Kaur J, Kumar K, Yadav AB, Misra A (2008). Intracellular time course, pharmacokinetics, and biodistribution of isoniazid and rifabutin following pulmonary delivery of inhalable microparticles to mice. *Antimicrobial Agents and Chemotherapy*.

[14] Weers J, Metzheiser B, Taylor G, Warren S, Meers P, Perkins WR (2009). A gamma scintigraphy study to investigate lung deposition and clearance of inhaled amikacin-loaded liposomes in healthy male volunteers. *Journal of Aerosol Medicine and Pulmonary Drug Delivery*.

[15] Jain SK, Gupta Y, Ramalingam L (2010). Lactose-conjugated PLGA nanoparticles for enhanced delivery of rifampicin to the lung for effective treatment of pulmonary tuberculosis. *PDA Journal of Pharmaceutical Science and Technology*.

[16] Wang J-S, Zhu J-B, Lü R-Q, Shen W (2005). Preparation of lung targeting azithromycin liposomes and its tissue distribution in mice. *Yaoxue Xuebao*.

[17] Xiang Q-Y, Wang M-T, Chen F (2007). Lung-targeting delivery of dexamethasone acetate loaded solid lipid nanoparticles. *Archives of Pharmacal Research*.

[18] Gutiérrez Millán C, Zarzuelo Castañeda A, González López F, Sayalero Marinero ML, Lanao JM (2008). Pharmacokinetics and biodistribution of amikacin encapsulated in carrier erythrocytes. *Journal of Antimicrobial Chemotherapy*.

[19] Briones E, Colino CI, Millán CG, Lanao JM (2009). Increasing the selectivity of amikacin in rat peritoneal macrophages using carrier erythrocytes. *European Journal of Pharmaceutical Sciences*.

[20] Xiang Q-Y, Wang M-T, Chen F (2007). Lung-targeting delivery of dexamethasone acetate loaded solid lipid nanoparticles. *Archives of Pharmacal Research*.

[21] Videira MA, Botelho MF, Santos AC, Gouveia LF, Pedroso De Lima JJ, Almeida AJ (2002). Lymphatic uptake of pulmonary delivered radiolabelled solid lipid nanoparticles. *Journal of Drug Targeting*.

